# Hormonal and electrolyte predictors for methotrexate versus surgery in ectopic pregnancy

**DOI:** 10.1371/journal.pone.0327435

**Published:** 2025-07-02

**Authors:** Yusuf Başkıran, Kazım Uçkan, İzzet Çeleğen, Fatma Başak Tanoğlu

**Affiliations:** 1 Istinye University Faculty of Medicine, Gynecology and Obstetrics Clinic, Istanbul, Turkey; 2 Van Yuzuncu Yil University Faculty of Medicine, Yuzuncu Yil University, Faculty of Medicine, Gynecology and Obstetrics Clinic, Van, Turkey; 3 Faculty of Medicine Department of Public Health, Yuzuncu Yil University, Van, Turkey; 4 Acibadem Healthcare Gruop, Gynecology and Obstetrics Clinic, Istanbul, Turkey; Shahid Beheshti University of Medical Sciences School of Medicine, IRAN, ISLAMIC REPUBLIC OF

## Abstract

This retrospective study investigates the association between maternal serum electrolyte and hormone levels and the success of methotrexate (MTX) treatment in ectopic pregnancies, aiming to identify predictive factors and improve clinical outcomes. A total of 372 patients treated with single-dose MTX between 2012 and 2023 were included, divided into two groups: those who successfully responded to MTX and those requiring surgical intervention. Significant differences were observed in beta-hCG levels, with a mean of 1376 in the MTX-successful group and 2358 in the surgical group. Electrolyte analysis showed that patients who responded successfully to MTX had higher potassium and albumin levels, whereas magnesium levels were lower compared to the surgical group. Haematological parameters also varied, with higher hemoglobin and hematocrit levels observed in the MTX-successful group. The findings suggest that serum potassium, magnesium, and albumin levels may be predictive markers for MTX efficacy. Higher potassium and albumin levels, along with lower magnesium levels, may contribute to improved treatment outcomes. These results support the potential role of dietary interventions—such as increasing potassium and albumin intake and moderating magnesium levels—in enhancing MTX success rates. This study underscores the importance of maternal biochemical profiles in guiding treatment strategies for ectopic pregnancy and highlights the value of individualized approaches to optimize outcomes and minimize the need for surgical intervention. Future research should aim to elucidate the biological mechanisms underlying these associations and validate the findings in larger and more diverse patient populations.

## Introduction

Ectopic pregnancy, characterized by the implantation of a fertilized ovum outside the uterus, represents a critical challenge in gynecology. Among various ectopic implantation sites, the fallopian tube, particularly the ampullary region, is the most frequent location [1]. This condition poses a significant risk to maternal health, with factors such as a history of ectopic pregnancy, pelvic inflammatory disease, smoking, previous tubal surgeries, and assisted reproductive technologies notably increasing its likelihood [2].

Advancements in diagnostic techniques, including the combined use of transvaginal ultrasonography and beta–human chorionic gonadotropin (beta-hCG) measurements, have significantly improved the early detection of ectopic pregnancies [3]. Early diagnosis plays a pivotal role in altering treatment approaches, enabling management before rupture occurs and thereby reducing mortality and morbidity [4].

If not diagnosed and managed in a timely manner, ectopic pregnancy may lead to serious complications such as tubal rupture, intra-abdominal bleeding, hypovolemic shock, and even maternal death, underscoring the importance of early risk stratification [5,6]. Despite these advances, the invasive nature of ectopic pregnancy often induces inflammatory responses, characterized by elevated levels of tumor necrosis factor (TNF)-alpha, interleukin-6, and interleukin-8, which may further complicate treatment outcomes [7].

Single-dose methotrexate (MTX) therapy has become the preferred non-surgical treatment for clinically stable cases of ectopic pregnancy [8]. Administered intramuscularly at a dose of 50 mg/m², MTX requires close follow-up, particularly when beta-hCG levels fail to decrease by at least 15% between days 4 and 7, which may necessitate a second dose in approximately 14–20% of cases [9]. Reported success rates for single-dose MTX range from 64% to 80%, and treatment efficacy is strongly influenced by initial beta-hCG levels, which ideally should be below 5000 mIU/mL in the absence of fetal cardiac activity [10–12]. Methotrexate functions as a folic acid antagonist, inhibiting dihydrofolate reductase and thereby disrupting DNA synthesis and cell proliferation [11]. However, its pharmacodynamic effectiveness may also be modulated by the presence of electrolytes and coenzymes involved in redox and enzymatic reactions. Elements such as magnesium and potassium play essential roles in cellular metabolism, and their imbalance may affect MTX efficacy [13]. Given these biochemical interactions, individual variations in maternal serum profiles may be key to predicting treatment outcomes.

This study aims to evaluate the relationship between maternal serum electrolyte levels and the outcomes of single-dose MTX therapy, particularly focusing on patients who required surgical intervention following treatment failure. By identifying potential predictive markers, the study seeks to contribute to the development of improved clinical management strategies for ectopic pregnancy.

## Materials and methods

This retrospective study included 372 patients diagnosed with ectopic pregnancy and treated with a single dose of methotrexate between 2012 and 2023 at the septic unit of the obstetrics clinic. The diagnosis of ectopic pregnancy was established based on a combination of transvaginal ultrasonography findings, clinical symptoms, and irregularly elevated beta-hCG levels. Patients were categorized into two groups: those who responded to methotrexate treatment and those who required surgical intervention.

Exclusion criteria were as follows: patients younger than 18 or older than 40 years, those who underwent emergency surgery due to a ruptured ectopic pregnancy, those who declined methotrexate treatment, and those with contraindications such as chronic illness, methotrexate allergy, active peptic ulcer, or breastfeeding.

Ethical approval for the study was obtained from the institutional ethics committee (approval number: 2023/08–02). The study was conducted in accordance with the principles of the Declaration of Helsinki. As this was a retrospective study, obtaining written informed consent from participants was not applicable. The ethics committee reviewed and approved the use of existing medical records for research purposes and waived the requirement for individual informed consent. Due to the retrospective nature of the study and limitations in the available clinical data, detailed information on potential confounding variables—such as dietary habits, lifestyle factors, and comorbidities—could not be comprehensively obtained or statistically adjusted for in the analysis.

All patient data were fully anonymized prior to analysis, and no identifiable personal information was accessed by the researchers at any point during or after data collection, ensuring strict confidentiality. Data were accessed solely for research purposes on April 15, 2023, following ethical approval.

### Statistical Analysis

All statistical analyses were conducted using SPSS Version 20.0 (IBM Corp., Armonk, NY, USA). Categorical variables were presented as frequencies and percentages, while continuous variables were summarized as means and standard deviations. The Shapiro-Wilk test was utilized to assess the normality of data distribution. Group comparisons for normally distributed continuous variables were performed using the independent t-test. A p-value of <0.05 was considered statistically significant.

## Results

A total of 372 patients diagnosed with ectopic pregnancy and treated with methotrexate were included in the study. Among these, 46 patients did not respond to the first two doses of methotrexate and subsequently required surgical intervention. The mean age of the participants was 31.00 ± 5.21 years.

Table 1 summarizes the maternal characteristics of the two groups. No statistically significant differences were observed in variables such as age, parity, gravida, abortion history, body mass index (BMI), gestational age, previous ectopic pregnancy, weight, or height.

**Table 1:** Distribution of groups according to maternal characteristics.

	Methotrexate (n = 326)		Surgical (n = 46)		
**Mean**	**SD**	**Mean**	**SD**	**p**
**Age**	31.33	4.81	30.41	5.73	0.15
**Gravida**	2.80	1.47	2.95	1.65	0.09
**Parity**	1.18	1.18	1.28	1.28	0.08
**Abortion**	0.75	0.80	0.77	1.24	0.39
**EG**	0.08	0.34	0.07	0.20	0.07
**Height (cm)**	162.95	6.63	163.50	7.14	0.11
**Weight (kg)**	67.60	10.32	68.32	8.52	0.17
**BMI (kg/m2)**	25.82	4.00	25.32	3.03	0.43
**Gestational week**	6.60	0.66	6.45	0.99	0.21

Abbreviations: SD; Standard deviation, BMI; Body mass index. Values in bold represent statistically significant results

Table 2 presents hemogram and coagulation parameters. Statistically significant differences were found in hemoglobin (HGB) and hematocrit (HCT) levels, both of which were lower in the surgical group (p < 0.005). In contrast, platelet count (PLT), activated partial thromboplastin time (aPTT), and prothrombin time (PT) values were significantly higher in the methotrexate group. Inflammatory markers such as neutrophils (NEU) and monocytes (MON) were elevated in the surgical group, whereas lymphocyte (LYM) levels were higher in the methotrexate group. No significant differences were noted for mean platelet volume (MPV) and fibrinogen levels between the groups.

**Table2:** Distribution of groups according to hemogram and coagulation parameters.

	Methotrexate (n = 326)		Surgical (n = 46)		
**Mean**	**SD**	**Mean**	**SD**	**p**
**WBC (10** ^**9**^ **/L)**	8.27	2.06	9.10	3.20	**0.002**
**HGB (g/dl)**	12.92	1.31	12.82	1.8	**0.001**
**HCT (%)**	38.75	3.53	36.09	5.93	**0.001**
**MCV (fl)**	83.80	6.42	85.18	4.44	**0.029**
**PLT (10** ^**3**^ **/μl)**	306.07	103.71	283.04	53.12	**0.018**
**MPV (fl)**	10.10	0.76	9.99	1.00	0.26
**NEU (%)**	5.28	1.82	6.27	3.19	**0.001**
**LYM (%)**	2.30	0.71	2.09	0.73	**0.008**
**MON (%)**	0.25	0.43	0.45	0.50	**0.001**
**APTT (sec)**	29.05	3.92	27.05	3.43	**0.001**
**PT (sec)**	14.20	1.25	13.55	1.16	**0.001**
**PT (%)**	1.00	0.00	1.00	0.00	cannot be computed
**INR**	93.35	12.24	100.18	14.07	**0.001**
**Fibrinogen**	326.48	72.63	332.82	74.64	0.42

Abbreviations: SD; Standard deviation, WBC; White Blood Cell, Hb; hemoglobin, HCT, hematocrit, MON; Monocyte ratio, MCV; mean corpuscular volüme, PLT; Platelet count, MPV; Mean platelet volüme, aPTT; Activated partial thromboplastin time, INR; International normalized ratio, PT; Prothrombin time Values in bold represent statistically significant results.

Table 3 displays hormone and electrolyte levels across the groups. Thyroid hormone levels, including thyroid-stimulating hormone (TSH), free triiodothyronine (fT3), and free thyroxine (fT4), did not differ significantly between the methotrexate and surgical groups. However, albumin and creatinine levels were significantly higher in the methotrexate group. Among electrolytes, potassium levels were approximately 7.5% higher in the methotrexate group (4.3 vs. 4.0 mg/L), while magnesium levels were about 4.5% lower (1.91 vs. 2.00 mg/L) compared to the surgical group. No significant differences were observed for sodium and calcium levels. Similarly, there were no significant differences in aspartate aminotransferase (AST), alanine aminotransferase (ALT), and lactate dehydrogenase (LDH) levels between the groups.

**Table3:** Distribution of groups according to hormone and electrolyte values.

Parameter	Methotrexate Mean	Methotrexate SD	Surgical Mean	Surgical SD	p value	% Difference
**TSH (U/L)**	2.33	1.83	2.59	1.73	0.17	−10.0
**fT3 (pmol/L)**	3.28	0.44	3.23	0.73	0.43	1.5
**fT4 (pmol/L)**	1.1	0.3	1.05	0.2	0.65	4.8
**BUN (mg/dL)**	10.8	3.09	10.36	2.25	0.15	4.2
**CRE (mg/dL)**	0.73	0.44	0.55	0.5	0.001	32.7
**Albumin (g/dL)**	45.2	2.98	39.05	3.1	0.001	15.7
**AST (IU/L)**	21.1	5.62	21.91	9.33	0.29	−3.7
**ALT (IU/L)**	20.05	8.25	19.95	11.24	0.92	0.5
**LDH (IU/L)**	195.55	40.97	209.09	40.4	0.02	−6.5
**Na (mg/L)**	139.2	2.27	139.18	1.78	0.93	0.0
**Ca (mg/L)**	9.51	0.66	9.45	0.58	0.36	0.6
**K (mg/L)**	4.3	0.15	4.0	0.0	0.03	7.5
**Mg (mg/L)**	1.91	0.15	2.0	0.0	0.03	−4.5

Abbreviations: SD, standard deviation; TSH, thyroid-stimulating hormone; fT3, free triiodothyronine; fT4, free thyroxine; BUN, blood urea nitrogen; CRE, creatinine; AST, aspartate aminotransferase; ALT, alanine aminotransferase; LDH, lactate dehydrogenase; Na, sodium; Ca, calcium; K, potassium; Mg, magnesium. Values in bold represent statistically significant results.

Beta-hCG levels also differed significantly between the groups, with a mean value of 1996 in the methotrexate-responsive group and 2558 in the surgical group. This disparity may be partially attributed to socioeconomic factors influencing patient compliance with methotrexate, a chemotherapeutic agent.

## Discussion

Ectopic pregnancy remains a critical gynecological condition, significantly contributing to maternal morbidity and mortality worldwide [5,6]. Methotrexate (MTX), a widely recognized non-surgical treatment option, is highly effective in stable and non-ruptured ectopic pregnancies. Unlike surgical methods, MTX preserves the ovarian reserve and supports future fertility, making it a preferred choice in eligible cases [14]. Global data reveal MTX success rates ranging from 65% to 95%, underscoring its therapeutic potential [15]. In the present study, an impressive efficacy rate of 87.6% was achieved, highlighting its effectiveness in clinical practice.

Comprehensive reviews have investigated the factors influencing methotrexate effectiveness in reducing maternal morbidity and mortality in ectopic pregnancies. Among these, beta-hCG levels are consistently identified as key predictors of treatment success.Although an absolute threshold for beta-hCG that guarantees therapeutic success or predicts failure has not been universally established, a value of 5000 IU/L is frequently employed as a clinical benchmark for assessing the potential risk of treatment failure [14].

Several studies have reported a significant association between elevated white blood cell (WBC) counts—an indicator of inflammation—and methotrexate failure. WBC levels have been identified as an independent risk factor and may help predict treatment response [15]. In the present study, beta-hCG levels were significantly lower in the successful treatment group (mean: 1996) compared to the surgical group (mean: 2558), reinforcing its predictive value.

Previous research has also emphasized the potential role of inflammatory markers such as neutrophil-to-lymphocyte ratio (NLR), platelet count (PLT), and platelet distribution width (PDW) in predicting methotrexate efficacy [16,17]. Consistent with the literature, our study observed higher WBC and lower PLT levels in patients requiring surgical intervention.

One study reported significantly lower hemoglobin (Hb) and hematocrit (Hct) levels in the methotrexate failure group, with no significant changes in MPV, WBC, PLT, NLR, or PLR [17]. In line with these findings, our results showed reduced Hb, Hct, WBC, and MCV values in the successful group, along with elevated PLT counts. MPV was not significantly different.

Albumin levels—known for their anti-inflammatory and nutritional significance—were also notably higher in the methotrexate group. These findings suggest that increasing serum albumin may be associated with improved methotrexate response, although the underlying mechanisms remain to be clarified.

Beyond potassium, magnesium, and albumin, other biochemical markers such as creatinine and lactate dehydrogenase (LDH) also showed significant differences. Creatinine levels were 32.7% higher in the methotrexate group, while LDH levels were 6.5% lower compared to the surgical group.

Although TSH levels did not differ significantly between groups, a slight elevation in the surgical group may reflect physiological stress or underlying thyroid dysregulation.

Taken together, the observed 15.7% higher albumin and 7.5% higher potassium levels in the methotrexate group may further support the predictive value of these markers for favorable treatment outcomes.

A prior study evaluating combined methotrexate and mifepristone therapy found no significant benefit; however, it noted a potential contraindication for the concurrent use of potassium chloride with methotrexate due to adverse effects on efficacy [18].

In our study, successful methotrexate treatment was also associated with lower magnesium and higher potassium levels. These findings suggest that elevated potassium levels prior to treatment may correlate with better response, although causality cannot be established.

Dietary patterns influencing potassium and magnesium balance may warrant further investigation as potential modifiers of treatment outcomes.Most studies in the literature focus on the relationship between methotrexate success and factors such as inflammation and beta-hCG levels. In contrast, this study investigates the influence of maternal electrolyte levels, providing new insights into potential predictors of methotrexate success in ectopic pregnancy management.

Beta-hCG serves as a critical parameter in determining the need for surgical intervention over medical treatment in ectopic pregnancy cases. While white blood cell (WBC) count, an inflammation marker, has recently been considered for similar purposes, its lack of specificity to ectopic pregnancy limits its utility, underscoring the need for further detailed investigations.

The majority of studies linking methotrexate success focus on beta-hCG levels, with limited exploration of other pathophysiological factors. In this study, a novel observation was made: elevated potassium levels positively correlated with methotrexate success, whereas higher magnesium levels were associated with treatment failure. These findings highlight the potential role of electrolyte balance in influencing the efficacy of methotrexate, suggesting a new avenue for optimizing treatment outcomesAlthough potassium and magnesium levels appear to be promising predictors, the biological mechanisms linking these electrolytes to methotrexate efficacy remain speculative. Further mechanistic studies are necessary to validate these associations and clarify their role in treatment planning.

### Limitations

This study has some limitations that should be noted. First, the retrospective design may have introduced selection bias, limiting the ability to infer causality. Second, the study was conducted in a single center, potentially reducing the generalizability of the findings to other populations and healthcare settings. Third, some potential confounding factors, such as dietary habits, lifestyle variables, and detailed comorbid conditions, were not comprehensively analyzed. Lastly, the relatively small number of patients requiring surgical intervention might have impacted the statistical power for detecting differences in some parameters. Future research should aim for prospective, multicenter designs with larger sample sizes to address these limitations and validate the findings.

## Conclusion

The findings of this study suggest that maternal levels of magnesium, potassium, and albumin, alongside beta-hCG, can serve as predictive markers for identifying patients who may require surgical intervention in ectopic pregnancy management. A diet enriched with potassium and albumin while limiting magnesium intake may potentially enhance the success of methotrexate therapy at the time of admission.

Unlike most studies in the literature that evaluate these parameters independently, our findings emphasize the importance of considering them collectively to provide more accurate predictions. Future research should focus on larger, multicenter studies to validate these results and further elucidate the underlying mechanisms, offering a clearer and more detailed understanding of their role in methotrexate efficacy.

## Supporting information

S1 Data (XLSX)

## References

[1] Committee on Practice Bulletins ACOG. Tubal ectopic pregnancy. Obstet Gynecol. 2018;131(2):e65–77. doi: 10.1097/AOG.0000000000002464 29232273

[2] Brady PC. New evidence to guide ectopic pregnancy diagnosis and management. Obstet Gynecol Surv. 2017;72(10):618–25. doi: 10.1097/OGX.0000000000000492 29059454

[3] Hendriks E, Rosenberg R, Prine L. Ectopic pregnancy: diagnosis and management. Am Fam Physician. 2020;101(10):599–606. 32412215

[4] Luciano AA, Roy G, Solima E. Ectopic pregnancy from surgical emergency to medical management. Ann N Y Acad Sci. 2001;943:235–54. doi: 10.1111/j.1749-6632.2001.tb03805.x 11594543

[5] Centers for Disease Control and Prevention (CDC). Ectopic pregnancy United States, 1990–1992. JAMA. 1995;273:533. 7837386

[6] Al Naimi A, Moore P, Brüggmann D, Krysa L, Louwen F, Bahlmann F. Ectopic pregnancy: a single-center experience over ten years. Reprod Biol Endocrinol. 2021;19(1):79. doi: 10.1186/s12958-021-00761-w 34059064 PMC8166577

[7] Rausch ME, Barnhart KT. Serum biomarkers for detecting ectopic pregnancy. Clin Obstet Gynecol. 2012;55(2):418–23. doi: 10.1097/GRF.0b013e31825109f6 22510623 PMC3329643

[8] Tonick S, Conageski C. Ectopic Pregnancy. Obstet Gynecol Clin North Am. 2022;49(3):537–49. doi: 10.1016/j.ogc.2022.02.018 36122984

[9] Kanmaz AG, Inan AH, Beyan E, Budak A. Role of various complete blood count parameters in predicting the success of single-dose Methotrexate in treating ectopic pregnancy. Pak J Med Sci. 2018;34(5):1132–6. doi: 10.12669/pjms.345.15356 30344563 PMC6191789

[10] Nadim B, Lu C, Infante F, Reid S, Condous G. Relationship Between Ultrasonographic and Biochemical Markers of Tubal Ectopic Pregnancy and Success of Subsequent Management. J Ultrasound Med. 2018;37(12):2899–907. doi: 10.1002/jum.14652 29675930

[11] Obaid M, Abu-Faza M, Abdelazim IA, Al-Khatlan HS, Al-Tuhoo AM. Undisturbed tubal pregnancies with positive fetal heart treated medically: Case study. J Mother Child. 2023;26(1):124–6. doi: 10.34763/jmotherandchild.20222601.d-22-00037 36803944 PMC10032323

[12] Bonin L, Pedreiro C, Moret S, Chene G, Gaucherand P, Lamblin G. Predictive factors for the methotrexate treatment outcome in ectopic pregnancy: A comparative study of 400 cases. Eur J Obstet Gynecol Reprod Biol. 2017;208:23–30. doi: 10.1016/j.ejogrb.2016.11.016 27888702

[13] Hudlicky M. Reductions in organic chemistry. 2nd ed. Washington, DC: American Chemical Society. 1996.

[14] The Practice Committee of the American Society for Reproductive Medicine. Medical treatment of ectopic pregnancy: a committee opinion. Fertility and Sterility. 2013;100(3):638–44. 23849842 10.1016/j.fertnstert.2013.06.013

[15] Zhang J, Zhang Y, Gan L, Liu X-Y, Du S-P. Predictors and clinical features of methotrexate (MTX) therapy for ectopic pregnancy. BMC Pregnancy Childbirth. 2020;20(1):654. doi: 10.1186/s12884-020-03350-8 33121473 PMC7597060

[16] Li Z, Yang F, Dunn S, Gross AK, Smyth SS. Platelets as immune mediators: their role in host defense responses and sepsis. Thromb Res. 2011;127(3):184–8. doi: 10.1016/j.thromres.2010.10.010 21075430 PMC3042496

[17] Kan Ö, Gemici A, Alkilic A, Cetindag EN, Cakir C, Dur R, et al. The effect of preoperative neutrophil-to-lymphocyte ratio and platelet-to-lymphocyte ratio on predicting rupture risk in tubal ectopic pregnancies. Gynecol Obstet Invest. 2019;84(4):378–82. doi: 10.1159/000496543 30654361

[18] Rozenberg P, Chevret S, Camus E, de Tayrac R, Garbin O, de Poncheville L, et al. Medical treatment of ectopic pregnancies: a randomized clinical trial comparing methotrexate-mifepristone and methotrexate-placebo. Hum Reprod. 2003;18(9):1802–8. doi: 10.1093/humrep/deg344 12923131

